# Meat Consumption and Depression: An Updated Systematic Review and Meta-Analysis

**DOI:** 10.3390/nu17050811

**Published:** 2025-02-26

**Authors:** Andrea Luque-Martínez, Ángel Francisco Ávila-Jiménez, Ángela Reinoso-Espín, Miguel Ángel Araújo-Jiménez, Cynthia Raquel Martos-Salcedo, Pablo González-Domenech, Sara Jiménez-Fernández, Virginia Martínez-Ruiz, Naomi Cano-Ibáñez, Mario Rivera-Izquierdo

**Affiliations:** 1 Service of Preventive Medicine and Public Health, Hospital Universitario San Cecilio, 18016 Granada, Spain; andrea.luque.martinez.sspa@juntadeandalucia.es; 2 Faculty of Medicine, University of Granada, 18016 Granada, Spain; angelfranaj@correo.ugr.es (Á.F.Á.-J.); angelareies@correo.ugr.es (Á.R.-E.); miguelarajim@correo.ugr.es (M.Á.A.-J.); cynthiamartos@correo.ugr.es (C.R.M.-S.); 3 Academia de Alumnos Internos (AAI), University of Granada, 18016 Granada, Spain; 4 Department of Psychiatry, University of Granada, 18016 Granada, Spain; pgdomenech@ugr.es (P.G.-D.); sjimenez@ugr.es (S.J.-F.); 5 Child and Adolescent Mental Health Unit, Virgen de las Nieves University Hospital, 18014 Granada, Spain; 6 Department of Preventive Medicine and Public Health, University of Granada, 18016 Granada, Spain; virmruiz@ugr.es (V.M.-R.); ncaiba@ugr.es (N.C.-I.); 7 Instituto Biosanitario de Granada (ibs.GRANADA), 18014 Granada, Spain; 8 CIBER de Epidemiología y Salud Pública (CIBERESP), 28029 Madrid, Spain

**Keywords:** vegetarianism, flexitarianism, diet, depression, social, meta-analysis

## Abstract

**Background**: Several original studies have reported an inconsistent association between low meat consumption (e.g., vegetarian diets) and the risk of depression. The aim of this study was to quantify the relationship between low meat consumption and depression, identifying possible sources of heterogeneity and the potential role of psychosocial variables. **Methods**: A systematic review and meta-analysis were performed and reported according to PRISMA guidelines through a comprehensive search in Medline, Web of Science, Scopus, and PsychInfo databases from inception to January 2024 (PROSPERO registration ID: CRD42023405426). The exposures analyzed were (1) a meat-free diet and (2) a flexitarian (low-meat) diet. The outcome was depression. The meta-analysis included twenty longitudinal observational studies. Forest plots were designed, and heterogeneity was analyzed through *I*^2^ statistic and subgroup analyses. Publication bias was assessed through funnel plots and Egger’s test. **Results:** The pooled overall analysis showed a protective association (HR: 0.74, 95%CI: 0.59–0.89, *I*^2^ = 53.9%) between meat-free consumption and depression, which was consistent in the group of highest-quality studies. The main sources of heterogeneity identified were study quality, study design, year and country of publication, gender inequality in the country, and adjustment for certain variables (including social variables). The association between flexitarian diet and depression (HR: 0.90, 95%CI: 0.81–0.99, *I*^2^ = 58.9%) was not consistent between subgroups. **Conclusions**: The results of this meta-analysis show a consistent protective association between meat-free diets and depression and an inconclusive association regarding flexitarian diet. Primary studies analyzing psychosocial variables are needed to explain these results.

## 1. Introduction

Several authors have warned about the risks to health [1,2,3] and the environment [4,5] involved in meat production and consumption, especially concerning red and processed meats. Metabolic syndrome, diabetes, coronary heart disease, stroke, colorectal cancer, and all-cause mortality are some of the outcomes consistently associated with meat consumption [2]. This resulted in recommendations from international organizations to reduce meat in diet [6]. Thus, according to the World Cancer Research Fund International (2018) [7], red meat consumption should be limited to three portions per week (350–500 g per portion), while consumption of processed meat should be very low or eliminated.

Nevertheless, some authors reported that low-meat or meat-free diets might increase the risk of certain conditions such as depression [8], which represents another important public health issue. In fact, depression is considered the leading cause of disability [9]. To date, this association has been mostly studied from a biomedical approach, ignoring the psychological, social, and spiritual aspects that could play a crucial role in the association under study. To the best of our knowledge, four previous systematic reviews and meta-analyses tried to specifically approach this association, with high heterogeneity in their pooled analyses. Two of them [8,10] showed an increased risk of depression in meat-free consumers, another study [11] reported a higher risk of depression in patients with a meat-rich dietary pattern, and another study reported no association between vegetarian diet and depression [12]. However, these reviews presented important limitations that were reported by the authors. For example, the failure to identify sources of heterogeneity, the absence of publication bias assessment, or the presence of very heterogeneous exposures (including meat-free consumption, low-meat dietary patterns, vegetarianism, or veganism simultaneously). More importantly, all of them included cross-sectional studies that prevent establishing a correct temporal sequence between exposure and outcome, thus raising the possibility of reverse causation bias (depression affecting meat diet). In addition, a lack of a psycho-social-spiritual analysis concerning whether some social aspects such as religion, culture, gender, or socioeconomic level could be affecting this association is lacking. We tried to overcome such limitations through the inclusion of only longitudinal studies, a deep analysis of potential sources of heterogeneity, and a biopsychosocial perspective. Given the limitations of previous research, the inconsistency of results, the identification of recent high-quality analytical studies that were not included in previous reviews, and the current uncertainty regarding the study association (in constant social and health debate), we consider the necessity of an updated rigorous systematic review and meta-analysis on this association.

The objective of this study was to quantify the association between meat-free or low-meat consumption and the development of depression according to longitudinal studies and to analyze potential sources of heterogeneity from a biopsychosocial perspective.

## 2. Materials and Methods

### 2.1. Design and Research Strategy

A systematic review and meta-analysis were performed using meat-free and low-meat consumption as exposure and the development of depression as an outcome. The protocol was prospectively registered in PROSPERO (University of York) (ID: CRD42023405426). PRISMA (Preferred Reporting Items for Systematic reviews and Meta-Analysis) [13] recommendations were followed to ensure the quality of the reported information. Medline, Web of Science, Scopus, and Psychinfo databases were searched from inception to January 2024. A very sensitive search strategy was designed to minimize the likelihood of missing relevant articles. The search equation was as follows: (vegan* OR plant-based OR “plant-based” OR herbivore OR vegetarian OR meat) AND (depress*) AND (cohort OR case-control OR “case-control” OR trial OR longitudinal OR analyst*). No filters related to language or publication date were applied.

Titles and abstracts were independently assessed by different researchers to exclude non-relevant studies. Duplicate articles were identified and eliminated. After that, a full-text assessment was conducted independently according to inclusion and exclusion criteria. A cluster (inverse) search, including an assessment of references from relevant articles, was performed to complete the search process. Inconsistencies were solved by consensus with an experienced researcher in this methodology.

### 2.2. Study Selection

The PICOS criteria used for inclusion and exclusion criteria are summarized in Table 1. The inclusion criteria for selecting the studies were: (1) studies considering zero (meat-free) or reduced (low-meat) consumption of meat as exposure. Vegetarian, vegan, or flexitarian diets were considered in this group, as well as those who analyzed meat intake alone (dietary patterns according to the quantity of meat consumed); (2) studies considering depression development as outcome, measured through clinical records, diagnosed from a mental health care professional or using validated scales for clinical depression, depressive symptoms, or depression risk; (3) observational longitudinal (cohort or case-control) or experimental design; (4) results reported as strength of association measurements, i.e., risk ratio (RR), odds ratio (OR) or hazard ratio (HR), with 95% confidence intervals or sufficient information to calculate it. The only exclusion criteria were the presence of repeated cohorts (those that better adjusted to inclusion criteria or, alternatively, the most recent cohort was selected) and the impossibility of access to the full-text document or relevant quantitative data after contacting authors for data requests.

### 2.3. Data Extraction

A detailed form was designed to extract relevant data from each study into a spreadsheet, which included:

- First author’s name;
- Cohort name;
- Study design (cohort or case-control, prospective or retrospective);
- Publication year;
- Country;
- Research team composition (inclusion of epidemiology or statistics experts);
- Median follow-up time;
- Study population type (general population, pregnant women, adolescents, etc.);
- Study population size;
- Number of exposed individuals;
- Number of cases;
- Type of exposure (meat-free diet, including vegetarian and vegan diets, and low-meat diets, including flexitarian diets);
- Exposure measurement (food frequency questionnaire, etc.);
- Outcome type (depression/risk of depression/depression symptoms);
- Outcome measurement type (scale/professional diagnosis/clinical record);
- Model fitting variables (those included in the model with the highest number of fitting variables);
- Social variables used in adjusted models (marital status, educational level, housing, employment, income, ethnicity, satisfaction with socioeconomic status, subjective health status, etc.);
- Statistical measurement of association strength (RR/OR/HR);
- Statistical value (estimator) and 95% confidence interval;
- Subsequent calculations were performed to obtain other secondary variables of interest, such as the Human Development Index (HDI), Gender Social Norms Index (GSNI) according to the United Nations Development Programme, life expectancy at birth, schooling, or Gross Domestic Product (GDP) per capita for each country in the study, based on the most recent data from the United Nations Development Programme).

The Newcastle-Ottawa Scale (NOS) was used to evaluate the risk of bias in the studies included in the systematic review and meta-analysis [14]. The NOS score measures the risk of bias based on the presence of potential selection, information, and confounding biases. According to the NOS score, the selected studies were categorized as high quality (8–9 stars), medium quality (6–7 stars), and low quality (5 stars or less). The scale was applied independently by the researchers, and disagreements were resolved by a senior researcher.

### 2.4. Meta-Analytic Techniques

Association measures were obtained from each of the selected studies included, along with their 95% confidence intervals. ORs were calculated when primary studies only provided the beta statistic. Likewise, when articles offered values regarding the relationship between very high meat consumption and depression, their inverse value was calculated. Odds ratios and relative risks provided by the studies were approximated to hazard ratios in line with other consulted meta-analyses [15].

Articles including results from different samples were treated as separate articles [16,17,18]. A random-effects model was used for statistical analysis, considering the expected heterogeneity among studies. Several forest plots summarizing the main findings of the meta-analysis were generated. To assess heterogeneity among the included studies, the *I*^2^ index and the *p*-value for Cochran’s Q test were calculated. Potential sources of heterogeneity were explored by stratifying studies based on relevant variables through subgroup analyses, which were in line with our theoretical framework and initial hypothesis. Studies were divided into two groups based on exposure: “meat-free diet” and “low-meat diet”. Subgroup analyses were again conducted to explore sources that could be generating heterogeneity within each group.

Similarly, publication bias was graphically analyzed through funnel plots and statistically quantified using Egger’s test for each of the two described groups. All statistical analyses were performed using Stata^®^ V.17 statistical software (Stata Corp^®^, College Station, TX, USA).

## 3. Results

### 3.1. Search and Selection Process

**Figure 1:**
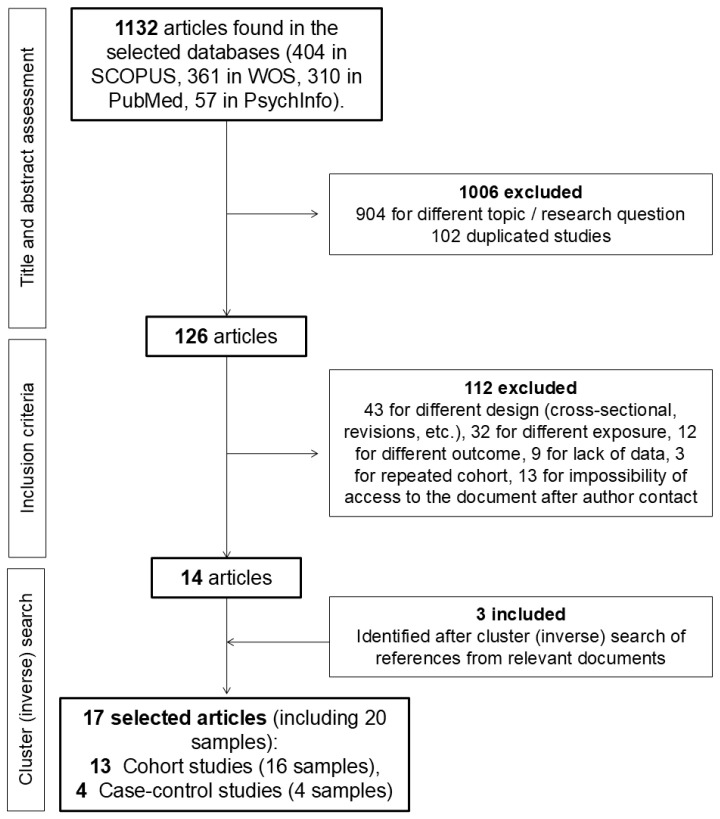
Flow chart of the study selection process.

Figure 1 summarizes the selection process of the articles included in the meta-analysis. The search strategy generated 1132 articles. After the assessment of the title and abstract of all articles, 126 were selected for full-text evaluation. Those articles that did not meet all the inclusion criteria were excluded. After this process, 14 papers, including 18 samples, were selected. This is due to the fact that the study by Lavallee et al. [17] stratified their findings between Chinese and German students (with no global results), while the studies by Hart et al. [16] and Northstone et al. [18] divided their results by sex.

Subsequently, a cluster (inverse) search was performed, evaluating articles included in other relevant studies (e.g., previous meta-analyses on this topic). In total, 3 articles were selected that met all the inclusion criteria. Thus, the study finally included 17 articles. Results for 2 different cohorts were provided by 3 of them, and the data for each cohort was recorded and analyzed separately so that the meta-analysis included 20 different samples from 17 published studies [16,17,18,19,20,21,22,23,24,25,26,27,28,29,30,31,32], 16 from cohort studies, and 4 from case-control studies.

### 3.2. Characteristics of the Studies and Quality Assessment

Table 2 summarizes the main characteristics of the selected studies. The included studies were published between the years 2009 and 2021, although 50% of them were published from 2017 onwards. The total number of participants in the meta-analysis was 65,339. The number of the exposed sample was only provided in 11 of the included studies. Counting these, the total number of exposed participants was 11,469 out of 39,175 (29.3% meat-free or low-meat consumers). In 14 of the included articles, the number of participants presenting depression or symptoms of depression at the end of follow-up was given, accounting for 3922 out of 36,118 (10.9%) participants. The rest of the characteristics of the selected articles can be consulted in detail in Table 2.

Regarding quality assessment (risk of bias) according to NOS, the results of the evaluation are presented in the (Supplementary Table S1). Briefly, 3 (15%) studies presented a high risk of bias, 4 (20%) showed a low risk of bias, and 13 (65%) were evaluated as medium quality (medium risk of bias).

### 3.3. Meat and Depression: Global Pooled Results

**Figure 2:**
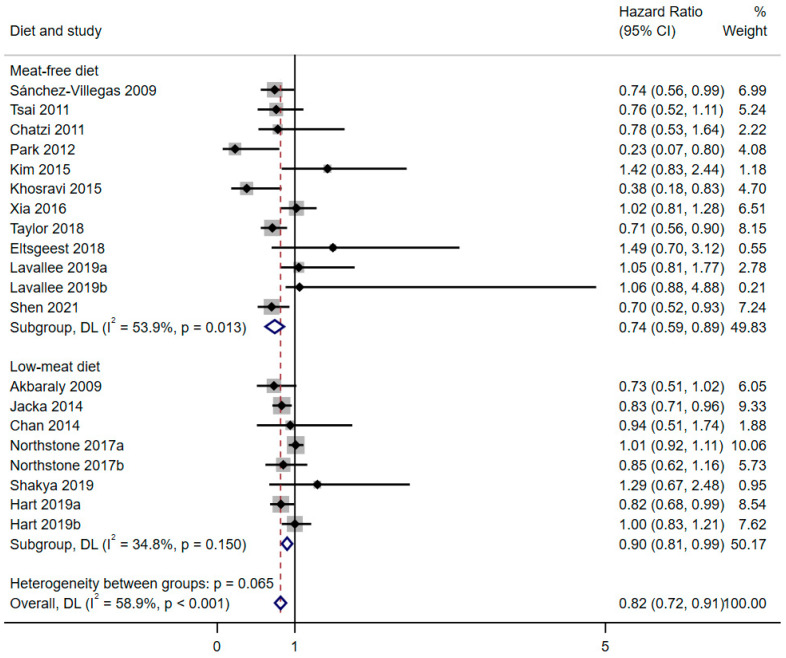
Forest plot of depression risk stratified by diet (meat-free or low-meat) [16,17,18,19,20,21,22,23,24,25,26,27,28,29,30,31,32].

The main analysis showed that diets with low consumption of meat (pooling meat-free and low-meat diet studies together) were protectively associated with the risk of depression (HR = 0.82; 95%CI: 0.72–0.91), with moderate heterogeneity (*I*^2^ = 58.9%; *p* = 0.065) (Figure 2). Given the heterogeneity obtained, subgroup analyses were conducted according to the most relevant variables in accordance with the theoretical framework and the hypothesis (Supplementary Table S2). For the remaining analysis presented in this meta-analysis, the sample was divided into two groups according to the type of diet (dietary assessment).

The horizontal line represents the length of the confidence interval, and the point value (estimator) is represented as a small black point within the interval. The size of the square for each study (grey shading) represents the weight of each study in the meta-analysis. The pooled values for each group (and for the total meta-analysis) are shown as a blue diamond. The horizontal length of the diamond represents the length of the pooled confidence interval

The “meat-free diet” included studies in which the exposure was zero meat consumption: vegetarian (or vegan) diet or no meat consumption [17,21,22,24,25,26,27,29,30,31,32]. The results obtained showed an inverse (protective) association between meat-free consumption and the risk of depression (HR = 0.74; 95%CI: 0.59–0.89), with moderate heterogeneity (*I*^2^ = 53.9%; *p* = 0.013). The “low-meat diet” group included 8 samples where the exposure was a dietary pattern obtained by “a posteriori” methods that compared lower-meat consumers with higher-meat consumers [16,19,20,23,28]. The results obtained for this group showed an inconclusive (and inconsistent between subgroups) association with the risk of depression, with an upper CI value close to the null value (HR = 0.90; 95%CI: 0.81–0.99) and moderate-low heterogeneity (*I*^2^ = 34.8%; *p* = 0.150). Figure 2 represents the forest plot stratified by meat-free and low-meat consumption subgroups.

### 3.4. Association Between Meat-Free Diet and Depression

Table 3 shows the results obtained for the subgroup analyses regarding a meat-free diet. The pooled HR for depression in cohort studies showed a protective effect (HR = 0.74; 95%CI: 0.64–0.84) with no heterogeneity (*I*^2^ = 0.0%), while case-control studies presented high heterogeneity (*I*^2^ = 85.2%), and a non-conclusive association with depression (HR = 0.70; 95%CI: 0.22–1.18). The result obtained for the subgroup analysis showed that the main sources of heterogeneity identified in this analysis were the study design (case-control designs), study quality according to the NOS scale (medium quality), year of publication (before 2018), area of publication (Asian countries), the presence of high gender inequality (>70% of the population according to the GSNI scale) in the country of the cohort, as well as the absence of adjustment of the models for previous depression, age, smoking, and for social variables (Table 3).

The results obtained for the subgroup analysis showed a tendency towards a more protective association between a meat-free diet and depression when more social variables were used for adjustments. However, some categories showed high heterogeneity. No potential publication bias was found by the visual assessment of the funnel plot and confirmed by Egger’s linear regression test (*p*-value = 0.785) (Supplementary Figure S1).

### 3.5. Association Between Low Meat Consumption and Depression

Table 4 shows the results of subgroup analyses conducted for a low-meat diet, including 8 articles. Because of the reduced study sample in this group and given that heterogeneity was acceptable (*I*^2^ = 34.8%; *p* = 0.150) (Figure 2), subgroup analyses were only conducted for the most relevant variables according to the theoretical framework and the initial hypothesis presented. No relevant or evident sources of heterogeneity were observed. Nevertheless, the area of publication (Europe), the low life expectancy of the country of publication, and the number of social variables for adjustments showed a tendency to increase the heterogeneity of pooled results. No potential publication bias was found by the visual assessment of the funnel plot and confirmed by Egger’s linear regression test (*p*-value = 0.470) (Supplementary Figure S1).

## 4. Discussion

This meta-analysis, conducted on 17 longitudinal studies comprising 64,992 individuals, showed a protective association between a meat-free diet and the risk of depression. This association was consistent for cohort studies, studies with higher quality, and the most recently published studies. Subgroup analysis by population psychosocial variables supported that the relationship between a meat-free diet and depression could be influenced by psychosocial variables. However, these results were not confirmed for the low-meat diet. Although also protective, we obtained inconclusive results since the upper value of the confidence interval was very close to the null value, showing low heterogeneity. The subgroup analysis for this diet showed no evident sources of heterogeneity. The graphical and statistical analysis of small-study effects showed no evidence of potential publication bias for any association.

Previous studies have reported an association between meat-free or low-meat consumption and the risk of depression, with heterogeneous results. No systematic review or meta-analysis of exclusively longitudinal studies has been conducted to date, excluding cross-sectional designs to eliminate potential reverse causation bias. We included only longitudinal studies, performed a deep analysis of potential sources of heterogeneity, and included a biopsychosocial perspective, thus overcoming the limitations of previous syntheses [8,9,10,11,12].

This work provides some relevant findings in a context where there is no consistency regarding this association according to previous scientific literature. The results of our study agree partially with those obtained by some previously performed meta-analyses [12,33], whereas they are not in agreement with the results of other literature reviews [8,34]. The present meta-analysis is, to our knowledge, the only one to find a conclusive and consistent protective association between meat-free consumption and the development of depression, as well as the only one that specifically addresses the relevance of social variables to accurately quantify this association.

Some studies that approached vegetarianism from a sociological and psychological point of view [35,36] reported results that could explain our findings. For example, they showed psychosocial and spiritual differences between omnivores, flexitarians, and meat-free diet consumers. Strictly, meat-free diet consumers usually present ethical motivations (animalistic or environmentalist), while people who maintain a low but flexible consumption tend to respond to “health” motivations, such as weight loss or suffering from a serious illness that would benefit from a healthier diet [35,36]. Cultural norms, socioeconomic status, concern for one’s health, the political-economic situation of the country, or religious practices are among the factors that could influence both diet and risk of depression. Therefore, if those psycho-socio-spiritual variables are not collected and considered, the association between meat consumption and mental health outcomes would be biased. Other studies that quantitatively analyzed the relationship between vegetarianism and depression [33,37] also support our findings. Both studies found an increased risk of depression in flexitarians compared to vegetarians (low-meat diet compared with meat-free diet). In our study, the evidence regarding low-meat consumption is weak due to borderline confidence intervals and the inconsistency of associations between subgroups. Hessler-Kaufman et al. (2021) focused on discerning whether the motivations for people adopting meat-free or flexitarian diets would be confounding the association between diet and depression [37]. Their results showed a consistent association between orthorexia (excessive preoccupation with healthy eating) and depression in the flexitarian group, which means that for flexitarians, orthorexia would confound the association between their diet and the risk of depression. Unfortunately, we could not verify this point since the primary studies did not include data on the reasons for diet election. In addition, it is worth considering the influence of Shen et al. (2021) on the protective association for depression found in the “meat-free diet” group [29]. This study was conducted in a Buddhist population where no animal consumption is well valued, but these results could not be applicable in other societies where meat-free diets are not socially valuable. Again, the influence of the sociocultural context becomes essential. The influence of some psychosocial variables could not be quantified in a meta-analysis because they were not collected in primary studies. Thus, gender could be acting as a confounder by being associated with both a meat-free diet [35,36] and depression [38]. Culture and social support are also considered relevant [35,36], as well as religion [29]. Similarly, socioeconomic status and other factors, such as personal motivation, could play a crucial role. Therefore, we believe that future primary studies should consider and collect these variables to obtain more accurate, unbiased results.

A thorough literature search strategy, clear inclusion and exclusion criteria, and quality assessment were performed in this study. The methods described here facilitate the reproducibility of this systematic review. Nevertheless, some limitations have to be considered. One of the main limitations of this study is related to the high heterogeneity of exposure measurement, which is frequent in dietary or nutritional studies. We tried to overcome this limitation by dividing the sample into two groups (meat-free and low-meat), depending on the methodology used to collect the diet. However, the low-meat group was composed of studies where the exposure was a dietary pattern constructed a posteriori, and this made it impossible to know the exact quantity of meat consumption. Besides, we grouped vegetarian and vegan diets under the term “meat-free” diet. Nevertheless, vegans are more likely to consume nutritional supplements that could be associated with a lower risk of depression [39,40]. Therefore, in our study, it is impossible to determine if the protective association is due to the exclusion of meat from the diet, the socio-psychological aspects associated with these diets, or the supplement intake. Regarding the outcome, the self-reported measurement of depression in many studies (validated scales completed by patients) might affect the results. Few of the included studies provided a diagnosis of depression made by a mental health professional [24,26,29], given that the use of scales for the diagnosis of depression or depressive symptoms in research is widespread. We conducted a subgroup analysis based on outcome assessment, and we found that meat-free consumption was associated with depression in the subgroup of studies with a diagnosis of depression (instead of self-reported symptoms). The quality assessment (NOS score) of the studies considers this point. In our meta-analysis, high-quality studies, which are less likely to use self-reported measures of exposure and outcome, support the general conclusions obtained in this meta-analysis. This study included only cohort and case-control studies. Case-control studies are more subject to selection and recall bias, which may have influenced our results, although only four case-control studies were included (all of them in the low-meat subgroup). We also included a subgroup analysis according to the study design. To our knowledge, this is the only meta-analysis on this association, including only longitudinal studies that make it possible to establish a temporal sequence between exposure (meat consumption) and the outcome (depression). No study from America or Africa matched the inclusion and exclusion criteria, so our results are unlikely to be applicable to these geographic areas. We also collected measures of association; given the absence of raw data from the original studies, we could not perform accurate meta-regression techniques. Finally, one of the aims of this meta-analysis was to study the potential influence of psychosocial variables on the association between meat consumption and depression. However, the lack of social perspective in most of the primary studies led to an absence of relevant social variables such as gender, socioeconomic status, culture, religion, and motivations for adopting a meat-free or low-meat diet.

The current context points towards the need to reduce global meat consumption and towards a global transformation of the food system [41]. Our results suggest the possibility that a meat-free diet could have positive consequences for mental health, protecting against depression. The abundant scientific evidence in favor of reducing meat consumption, together with our results, could encourage laws and government actions that facilitate the transition of the food system towards more local and seasonal consumption. That would lead to a reduction in exports, food waste, and the consumption of animal products, including meat. Although some scientific initiatives [41] and policies proposed in recent years advance along these lines, these have not materialized in real changes in meat consumption to date. Motivation to make a dietary change in this regard could be important and should be considered when formulating policy and health strategies to reduce dietary meat consumption. It is necessary that future primary original research studies attempt to analyze this association using a longitudinal observational design that allows for establishing a clear temporal association between exposure and outcome. In addition, collecting and adjusting for psychosocial variables, as well as concrete, validated, and standardized definitions of both exposure and outcome, could improve the degree of evidence on the association of interest.

## 5. Conclusions

In conclusion, a meat-free diet protected against depression in this meta-analysis. The low-meat diet did not provide conclusive results. Psychosocial variables influence this association, and future studies should analyze them. Analysis of study quality, sources of heterogeneity, and publication bias supports that the overall associations are consistent. More research is needed in this regard, including relevant socio-psychological variables, to corroborate our results and deeply understand the mechanisms behind dietary habits and mental health outcomes.

## Figures and Tables

**Table 1:** PICOS criteria for inclusion and exclusion of studies.

Parameter	Inclusion Criteria	Exclusion Criteria
Population	General population (human)	In vitro, other animal models
Intervention/exposure	Meat-free or low-meat diet	Other diets not stratified by meat consumption, healthy diets with no meat quantification
Comparator	Meat consumption or high-meat diet	No quantification of meat
Outcomes	Depression (through clinical records, professional diagnosis, or validated scales)	Non-validated diagnostic tools, other mental health diagnoses (e.g., anxiety)
Study design	Experimental (trial) or observational longitudinal (cohort or case-control)	Reviews, cross-sectional, conference abstracts, editorial and opinion pieces

**Table 2:** Characteristics of the studies included in this systematic review and meta-analysis.

Reference	Country	Population Characteristics	Sample Size (Total *n* = 64,992)	Exposure (Dietary) Assessment	Outcome (Depression) Assessment	Variables Used for Adjustments	NOS Score ^1^
Cohort studies							
Taylor, 2018 [30]	Australia	Multiple sclerosis patients	1401	Meat-free diet (no meat consumption)	PHQ-2 and PHQ-9 scales	Age, P-MSSS, FSS, antidepressant medication	Low (5)
Lavallee 2019a [17]	China	Students in China	12,744	Vegetarian diet	DASS-21 scale	None	Low (3)
Lavallee 2019b [17]	Germany	Students in Germany	1608	Vegetarian diet	DASS-21 scale	None	Low (3)
Eltsgeest 2018 [22]	Italy	Adults	757	Meat-free diet (no meat consumption)	CES-D scale	Age, sex, marital status, educational level, physical activity, smoking, BADL, alcohol consumption, and energy consumption.	Medium (7)
Sánchez-Villegas 2009 [27]	Spain	University students	10,094	Meat-free diet (no meat consumption)	Self-reported medical diagnosis or antidepressant consumption	Sex, age, smoking, energy intake, employment Age, sex, marital status, smoking, energy consumption, employment	High (8)
Tsai 2012 [31]	Taiwan	Taiwanese elderly	1609	Meat-free diet (no meat consumption)	CES-D10 scale	Sex, age, years of formal education, satisfaction with economic status, housing tenure, smoking, alcohol consumption, areca nut consumption, functional status, physical activity, cognitive status, presence of chronic comorbidities	Medium (7)
Akbaraly 2009 [19]	United Kingdom	Middle age white European adults	3486	Flexitarian diet	CES-D scale	Age, sex, energy intake, marital status, employment, education, physical activity, smoking, chronic diseases	Medium (6)
Shen 2021 [29]	Taiwan	Volunteers in the Buddhist foundation “Tzu Chi”	10,577	Vegetarian diet	At least two outpatient or one inpatient admission to the psychiatric department with a diagnosis of depression or dysthymia	Age, sex, educational level, marital status, physical activity, smoking, alcohol consumption, comorbidities.	High (8)
Chatzi 2011 [21]	Greece	Pregnant women	529	Meat-free diet (no meat consumption)	EPDS scale	Maternal age, maternal education, parity, housing, depression in previous pregnancies, energy intake during pregnancy	Medium (6)
Shakya 2019 [28]	Australia	Australian adults	859	Flexitarian diet	CES-D scale	Sex, age, energy intake, marital status, educational level, employment, income, socioeconomic index by area, alcohol consumption, smoking, physical activity, sleep quality, BMI, pain, hypertension, diabetes, CV disease	Medium (7)
Jacka 2014 [23]	Australia	Australian adults	3663	Flexitarian diet	“Goldberg” depression scale	Sex, phase of study, depressive symptoms at baseline	Medium (7)
Hart 2019a [16]	Australia	Australian women	1090	Flexitarian diet	Geriatric depression scale	Age, BMI, leisure time and physical activity, smoking, educational level, etc.	Medium (6)
Hart 2019b [16]	Australia	Australian men	1052	Flexitarian diet	Geriatric depression scale	Age, leisure time and physical activity, pain, smoking, educational level	Medium (6)
Chan 2014 [20]	China	Chinese elderly	2211	Flexitarian diet	Geriatric depression scale	Age, sex, energy intake, BMI, PASE, number of BADL, smoking, alcohol, education, marital status, history of diabetes, hypertension, heart disease and stroke, depression at baseline	Medium (7)
Northstone 2017a [18]	United Kingdom	Parenting women	6318	Flexitarian diet	EPDS scale	Age, educational level, ethnicity, housing, marital status, subjective health status, excessive number of cohabitants, anxiety score	High (8)
Northstone 2017b [18]	United Kingdom	Parenting men	2746	Flexitarian diet	EPDS scale	Age, educational level, ethnicity, housing, marital status, subjective health status, excessive number of cohabitants, anxiety score	High (8)
Case-control studies							
Park 2012 [26]	South Korea	Depressed patients in hospital	168	Meat-free diet (no meat consumption)	CES-D scale and medical interview	Alcohol consumption, marital status, hours of sleep, educational level, employment, etc.	Medium (6)
Kim 2015 [25]	South Korea	Adolescent women	849	Meat-free diet (no meat consumption)	K-BDS scale	Menstrual regularity and energy intake	Medium (6)
Khosravi 2015 [24]	Iran	Iranian adults	529	Vegetarian diet	Psychiatric interview (DSM-IV)	Non-antidepressant drug use, employment, BMI, number of children, marital status	Medium (6)
Xia 2016 [32]	China	Chinese adults	2702	Meat-free diet (no meat consumption)	ZSDS scale	Other food groups’ consumption	Medium (6)

BADL, basic activities of daily living; BMI, Body mass index; CES-D, Center for Epidemiologic Studies Depression Scale; CV, cardiovascular; DASS-21, Depression Anxiety and Stress Scale—21 items; DSM-IV, Diagnostic and Statistical Manual of Mental Disorders 4th edition; EPDS, Edinburg postnatal depression scale; FSS, Fatigue severity score; K-BDS, Korean Beck depression scale; PASE, Physical activity scale for the elderly; PHQ-2 and PHQ-9, Physical health quality 2 and 9 items; P-MSS, Patient-reported multiple sclerosis score; ZSDS, Zung depression scale. ^1^ Quality of studies according to NOS (Newcastle-Ottawa) scale: high quality (8–9 stars), medium quality (6–7 stars), and low quality (5 stars or less).

**Table 3:** Subgroup analyses for the association between a meat-free diet and risk of depression.

Subgroups	Studies (*n*)	HR (95%CI)	*p*-Value of Heterogeneity	*I*^2^ (%)
Study design				
Cohort	8	0.74(0.64–0.84)	0.838	0.0%
Case-control	4	0.70 (0.22–1.18)	0.000	85.2%
The age group of the population				
Students/Young adults	4	0.88 (0.62–1.15)	0.309	16.5%
All age groups	6	0.65 (0.45–0.86)	0.004	71.1%
Elderly	1	0.76 (0.46–1.05)	-	0.0%
Outcome				
Diagnosed depression	7	0.66 (0.45–0.87)	0.022	59.4%
Depressive symptoms	5	0.85 (0.70–1.00)	0.342	11.3%
Population selected with any other condition different from depression				
No	8	0.80 (0.62–0.98)	0.047	50.9%
Yes	4	0.64 (0.36–0.92)	0.048	62.1%
Gross Domestic Product per capita of the country				
<30,000$	6	0.77 (0.58, 0.97)	0.040	57.0%
≥30,000$	6	0.71 (0.45, 0.96)	0.045	55.9%
Human Development Index of the country				
<0.8	9	0.74 (0.57–0.91)	0.008	59.6%
≥0.8	3	0.86 (0.25–1.48)	0.207	37.2%
Study quality (NOS)				
High	2	0.72 (0.37–0.87)	0.792	0.0%
Medium	7	0.75 (0.45–1.05)	0.001	72.6%
Low	3	0.75 (0.59–0.91)	0.405	0.0%
Publication year				
<2015	4	0.63 (0.39–0.88)	0.090	53.7%
2015–2018	5	0.83 (0.53–1.13)	0.007	71.9%
>2018	3	0.76 (0.57–0.94)	0.577	0.0%
Area				
Europe	4	0.77 (0.57–0.96)	0.678	0.0%
Asia	7	0.74 (0.50–0.97)	0.001	72.9%
Australia	1	0.71 (0.54–0.89)	-	0.0%
Life expectancy in the country				
<81 years	7	0.77 (059–0.96)	0.069	48.8%
≥81 years	5	0.71 (0.44–0.89)	0.013	53.9%
Gender inequality in the country, according to the Gender Social Norms Index				
>70% of the population	7	0.74 (0.50–0.97)	0.001	72.9%
30–70% of the population	2	0.77 (0.57–0.96)	0.488	0.0%
<30% of the population	2	0.71 (0.54–0.88)	0.733	0.0%
Adjusted for employment				
No	8	0.85 (0.70–1.00)	0.191	29.7%
Yes	4	0.55 (0.31–0.80)	0.037	64.6%
Adjusted for marital status				
No	8	0.82 (0.71–0.94)	0.320	14.1%
Yes	4	0.52 (0.21–0.83)	0.039	64.2%
Adjusted for sex				
No	9	0.74 (0.53–0.95)	0.004	64.1%
Yes	3	0.73 (0.59–0.89)	0.013	53.9%
Adjusted for previous depression				
No	10	0.75 (0.56–0.94)	0.005	62.1%
Yes	2	0.72 (0.59–0.89)	0.013	0.0%
Adjusted for age				
No	6	0.77 (0.38–1.16)	0.001	77.4%
Yes	6	0.73 (0.63–0.83)	0.885	0.0%
Adjusted for alcohol consumption				
No	8	0.80 (0.62–0.97)	0.046	51.1%
Yes	4	0.63 (0.35–0.92)	0.053	61.0%
Adjusted for educational level				
No	5	0.82 (0.54–1.09)	0.015	67.7%
Yes	7	0.68 (0.52–0.89)	0.135	38.6%
Adjusted for smoking				
No	8	0.74 (0.50–0.99)	0.002	68.5%
Yes	4	0.74 (0.60–0.87)	0.013	0.0%
Adjusted for any social variable				
No	5	0.93 (0.70–1.16)	0.123	44.8%
Yes	7	0.63 (0.46–0.80)	0.013	46.6%
Number of social variables used for adjustments				
None	5	0.93 (0.70–1.16)	0.123	44.8%
One	1	0.74 (0.52–0.96)	-	0.0%
Two	2	0.71 (0.52–0.90)	0.791	0.0%
Three	4	0.54 (0.20–0.68)	0.043	63.2%

**Table 4:** Subgroup analyses for the association between low-meat diet and risk of depression.

Subgroups	Studies (*n*)	HR (95%CI)	*p*-Value of Heterogeneity	*I*^2^ (%)
Study quality (NOS)				
High	2	0.98 (0.86–1.10)	0.273	16.7%
Medium	6	0.85 (0.77–0.93)	0.484	0.0%
Area				
Europe	3	0.90 (0.72–1.08)	0.090	58.5%
Asia	1	0.94 (0.32–1.55)	-	0.0%
Australia	4	0.87 (0.77–0.97)	0.333	12.0%
Human Development Index of the country				
<0.8	7	0.90 (0.80–0.99)	0.097	44.1%
≥0.8	1	0.94 (0.32–1.55)	-	0%
Life expectancy in the country				
<81 years	4	0.91 (0.76–1.06)	0.185	37.8%
≥81 years	4	0.87 (0.77–0.97)	0.333	12.0%
Gross Domestic Product per capita of the country				
<30.000$	1	0.94 (0.32–1.55)	-	0.0%
≥30.000$	7	0.90 (0.80–0.99)	0.097	44.1%
Number of social variables used for adjustments				
None	1	0.83 (0.70–0.95)	-	0.0%
One	2	0.90 (0.73–1.06)	0.150	51.7%
Two	1	0.94 (0.48–0.99)	-	0.0%
Three	1	0.73 (0.48–0.99)	-	0.0%
Six	3	1.00 (0.91–1.06)	0.447	0.0%
Adjustments for any social variable				
None or one	3	0.87 (0.77–0.97)	0.278	21.9%
Two or more	5	0.93 (0.79–1.06)	0.256	24.8%
Adjustments for sex				
No	4	0.94 (0.83–1.04)	0.172	40%
Yes	4	0.82 (0.71–0.93)	0.640	34.8%
Adjustments for age				
No	1	0.83 (0.70–0.95)	-	0.0%
Yes	7	0.92 (0.82–1.02)	0.212	28.4%

## Data Availability

All data used in this study is reported within the manuscript and their Supplementary Files. The database can be shared upon request to the corresponding author.

## Supplementary Materials

The following supporting information can be downloaded at https://www.mdpi.com/article/10.3390/nu17050811/s1. Table S1: Evaluation of the selected studies according to Newcastle-Ottawa scale (NOS); Table S2: Subgroup analysis of the global results between low consumption of meat (pooling meat-free and low-meat diets) and risk of depression; Figure S1: Assessment of potential publication bias (small-study effect) through funnel plots and Egger’s test.

## Abbreviations

The following abbreviations are used in this manuscript:

GDP	Gross Domestic Product
GSNI	Gender Social Norms Index
HDI	Human Development Index
HR	Hazard ratio
NOS	Newcastle-Ottawa Scale
OR	Odds ratio
RR	Risk ratio
