# Supplementary material for: Meat Consumption and Depression: An Updated Systematic Review and Meta-Analysis
Source: Nutrients. 2025 Feb 26;17(5):811. doi: 10.3390/nu17050811 (PMC11901745; doi:10.3390/nu17050811)

Supplementary Table S1. Evaluation of the selected studies according to Newcastle-Ottawa scale (NOS).

Cohort studies	Total	S1	S2	S3	S4	Selection (S1-S4)	C	Comparability (C)	O1	O2	O3	Outcome (O1-O3)	Quality
Taylor 2018	5	1	1	0	1	3	1	1	0	1	0	1	Low
Lavallee 2019a	3	1	1	0	0	2	0	0	0	1	0	1	Low
Lavallee 2019b	3	1	1	0	0	2	0	0	0	1	0	1	Low
Eltsgeest 2018	7	1	1	1	1	4	2	2	0	1	0	1	Medium
Sánchez-Villegas 2009	8	1	1	1	1	4	2	2	0	1	1	2	High
Tsai 2011	7	1	1	1	1	4	2	2	0	1	0	1	Medium
Akbaraly 2009	6	0	1	1	1	3	2	2	0	1	0	1	Medium
Shen 2021	8	0	1	1	1	3	2	2	1	1	1	3	High
Chatzi 2011	6	1	1	1	1	4	2	2	0	0	0	0	Medium
Shakya 2019	7	1	1	1	1	4	2	2	0	1	0	1	Medium
Jacka 2014	7	1	1	1	1	4	2	2	0	1	0	1	Medium
Hart 2019a	6	1	1	1	0	3	2	2	0	1	0	1	Medium
Hart 2019b	6	1	1	1	0	3	2	2	0	1	0	1	Medium
Chan 2014	7	0	1	1	1	3	2	2	0	1	1	2	Medium
Northstone 2017a	8	1	1	1	1	4	2	2	1	1	0	2	High
Northstone 2017b	8	1	1	1	1	4	2	2	1	1	0	2	High
**Case-control studies**	**Total**	**S1**	**S2**	**S3**	**S4**	**Selection (S1-S4)**	**C**	**Comparability (C)**	**E1**	**E2**	**E3**	**Exposure (E1-E3)**	**Quality**
Park 2012	6	1	1	0	1	3	2	2	0	1	0	1	Medium
Kim 2015	6	0	1	1	1	3	2	2	0	1	0	1	Medium
Khosravi 2015	6	1	1	1	1	4	1	1	0	1	0	1	Medium
Xia 2016	6	0	1	1	1	3	1	1	1	1	0	2	Medium

Supplementary Table S2. Subgroup analysis of the global results between low consumption of meat (pooling meat-free and low-meat diets) and risk of depression.

Subgroups		Number of studies	Pooled results	Heterogeneity (p-value)	Heterogeneity (I^2)
Number of social variables used for adjustments					
	0	6	0.86 (0.73-1.00)	0.199	31.5%
	1	3	0.86 (0.71-1.00)	0.171	43.4%
	2	3	0.73 (0.55-0.91)	0.755	0.0%
	3	5	0.58 (0.32-0.84)	0.042	59.6%
	6	3	1.00 (0.91- 1.08)	0.447	0.0%
Meat-free diet		12	0.74 (0.59-0.89)	0.013	53.9%
Low-meat diet		8	0.90 (0.81-0.99)	0.150	34.8%
Europe		7	0.87 (0.73-0.01)	0.145	37.1%
Asia		8	0.75 (0.53-0-97)	0.002	69.0%
Australia		5	0.84 (0.74-0.91)	0.200	58.9%

Supplementary Figure S1. Assessment of potential publication bias (small-study effect) through funnel plots and Egger's test.

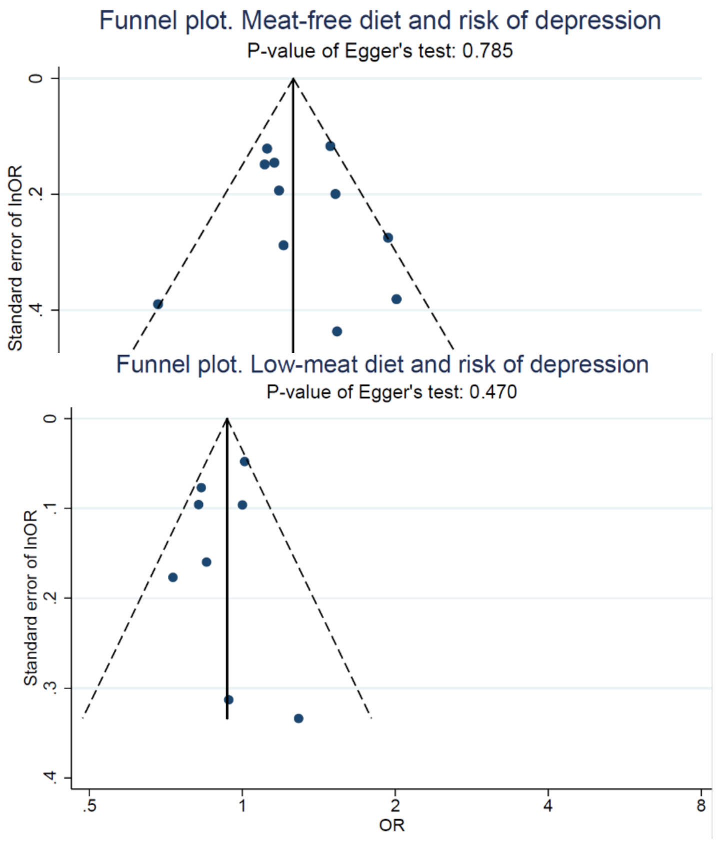

Supplement: Supplementary file 1 [file nutrients-17-00811-s001.zip › nutrients-3506396-supplementary.pdf]
